# Room Temperature Magnetic Memory Effect in Nanodiamond/γ-Fe_2_O_3_ Composites

**DOI:** 10.3390/nano11030648

**Published:** 2021-03-07

**Authors:** Ashish Chhaganlal Gandhi, Rajakar Selvam, Chia-Liang Cheng, Sheng Yun Wu

**Affiliations:** Department of Physics, National Dong Hwa University, Hualien 97401, Taiwan; acg.gandhi@gmail.com (A.C.G.); raju.rajkar@gmail.com (R.S.); clcheng@gms.ndhu.edu.tw (C.-L.C.)

**Keywords:** magnetic memory effect, magnetic nanodiamond, γ-Fe_2_O_3_, superparamagnetic, non-interacting

## Abstract

We report a room temperature magnetic memory effect (RT-MME) from magnetic nanodiamond (MND) (ND)/γ-Fe_2_O_3_ nanocomposites. The detailed crystal structural analysis of the diluted MND was performed by synchrotron radiation X-ray diffraction, revealing the composite nature of MND having 99 and 1% weight fraction ND and γ-Fe_2_O_3_ phases, respectively. The magnetic measurements carried out using a DC SQUID magnetometer show the non-interacting superparamagnetic nature of γ-Fe_2_O_3_ nanoparticles in MND have a wide distribution in the blocking temperature. Using different temperature, field, and time relaxation protocols, the memory phenomenon in the DC magnetization has been observed at room temperature (RT). These findings suggest that the dynamics of MND are governed by a wide distribution of particle relaxation times, which arise from the distribution of γ-Fe_2_O_3_ nanoparticle size. The observed RT ferromagnetism coupled with MME in MND will find potential applications in ND-based spintronics.

## 1. Introduction

The magnetic memory effect (MME) in various nanomaterials such as superparamagnetic (SPM) and the spin-glass (SG) system have been investigated intensively due to their complexity [1,2,3]. The non-interacting SPM system exhibits field cooled (FC) MME due to distribution in their relaxation times, which arises through particle size distribution. Whereas the SG system shows both FC and zero field cooled (ZFC) MME because of the surface effects, interparticle interactions, and the random distribution of the anisotropic axis. MME has been utilized as a fingerprint test in distinguishing SPM and SG systems [2]. For instance, the ferrimagnetic γ-Fe_2_O_3_ nanostructure having non-negligible interparticle interactions exhibits a superspin-glass (SSG) state where superspin freeze collectively into an SG-like state below a critical temperature. Such an SG-like system having a distribution of particle size exhibits both FC and ZFC MME [4,5]. Whereas only FC MME is reported from γ-Fe_2_O_3_/alginate nanocomposite, clearly demonstrating the effect of reduced interparticle interactions (i.e., reduced agglomeration) on the magnetic properties [6]. However, so far, the appearance of only low-temperature MME from γ-Fe_2_O_3_ far below the room temperature (RT) has hindered its use in composite materials for a potential application. In the past, the RT MME has been achieved through introducing additional magnetic anisotropy either by exchange-coupling, particle size distribution, or the inter-/intra-particle interactions [7,8,9,10].

In this study, we report RT MME from nanodiamond (ND)/γ-Fe_2_O_3_ composite nanoparticles coined as magnetic nanodiamonds (MNDs). NDs are a widely used potential material in biological and electronic applications and quantum engineering because of their low cost, high thermal conductivity, surface chemistry, biocompatibility, low toxicity, optical, electrical, and superconducting properties [11,12,13,14,15,16,17]. For a thorough structural investigation and to estimate the weight fraction of the constituent phases in MND synchrotron radiation powder X-ray diffraction (PXRD) facility was utilized, which is otherwise quite difficult to characterize using usual XRD techniques. For a deeper understanding of the magnetic properties, field, temperature, and time-dependent magnetization measurements were carried out using a DC superconducting quantum interference device (SQUID) vibrating sample magnetometer (VSM). Our findings suggest a drastic reduction in the interparticle interaction between γ-Fe_2_O_3_ because of the surrounding nonmagnetic NDs giving rise to more like an SPM behavior. The observed RT MME is discussed based on the multi-distribution of energy barriers aroused from the distribution of SPM γ-Fe_2_O_3_ particle sizes.

## 2. Materials and Methods

The MND powder sample was obtained from Ray Techniques Ltd., Jerusalem, Israel (RayND-M, Nanodiamond powder of laser synthesis modified, the metal-free, ferromagnetic). The as-received MND powder (without any further treatment) was utilized to perform structural characterization and the MME measurements. Before measurements material was stored in a dry box. Our experimental findings show that the MND is composed of ~90% ND and ~1% γ-Fe_2_O_3_ that will be discussed further in the text.

The synchrotron radiation PXRD facility at the National synchrotron radiation research center (NSRRC), Hsinchu, Taiwan (TLS 01C2 beamline, λ = 0.77491 Å, detector Mar345, measurement time ~5 min, beamline source is SWLS (superconductivity wavelength shift), and Flux (brilliance) is 1 × 10^11^) was utilized for the structural characterization of MND. The magnetic field, temperature, time-dependent magnetization, and the MME measurements were carried out using a DC SQUID magnetometer (Quantum Design, SQUID-VSM Ever Cool, San Diego, CA, USA). During temperature-dependent magnetic measurements, it was noticed that the magnetic properties of MND get slightly altered after reaching a measuring temperature of 400 K (~127 °C), whereas it retains the same properties at 350 K, possibly due to phase transition. Hence, before various measurements, the demagnetization of MND was performed at 350 K by initially setting a magnetic field to zero using inbuild oscillatory mode and then allowing the system to relax at the same temperature for 100 s. In oscillate mode, the field oscillates, in decreasing amplitude oscillations, around the setpoint eliminating the flux motion in the superconducting magnet windings. True zero field is best achieved using oscillate mode [18].

## 3. Results

### 3.1. Structural Properties

**Figure 1:**
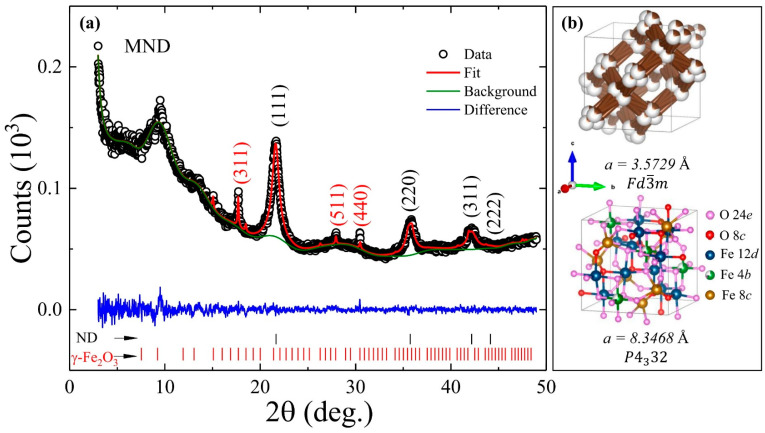
(**a**) The Rietveld refined (red line) powder X-ray diffraction (PXRD) diffractogram (open dots) of magnetic nanodiamond (MND). The green and blue lines represent the background and the difference between experimental and fitted diffractogram. The vertical black and red lines at the bottom of the figure mark Bragg’s positions for ND and γ-Fe_2_O_3_ phases, respectively. (**b**) A unit cell of ND and γ-Fe_2_O_3_ (top to bottom) with fitted values of lattice constant and the space group.

Figure 1a shows the PXRD diffractogram (open dots) from the MND sample composed of diffraction peaks from cubic ND and γ-Fe_2_O_3_ phases assigned based on space group $Fd\bar{3}m$ (No. 227) and $P4_{3}32$ (No. 212), respectively. A broad hump appeared around 2θ~9.5° can be assigned to graphite (002) [19]. The diffraction peaks from the ND phase are quite intense and broadened as compared to that of γ-Fe_2_O_3_. Using Scherrer’s formula, the estimated grain size from the most intense (111) and (311) diffraction peaks of ND and γ-Fe_2_O_3_ nanostructures is 4.74 nm and 43 nm, respectively. For further detailed structural characterization, Rietveld refinement (red line) of the PXRD diffractogram was carried out using a general structural analysis system (GSAS-II) software (red curve in Figure 1a) [20]. During the refinement process, crystallographic independent atomic coordinates and the occupancies at C and Fe 4*b* sites of ND and γ-Fe_2_O_3_ phases were kept free. The corresponding refinement parameters are summarized in Table 1. A lattice constant of 3.5729(12) Å and 8.3468(14) Å is obtained for ND and γ-Fe_2_O_3_, respectively, and the corresponding unit cells are depicted in Figure 1b. The fitted values of the lattice constant matches very well with the reported values in the literature [21,22]. Furthermore, the lattice constant of the ND phase in MND also matches very well with the acidic washed ND sample (see Table 1). A strong preference of the vacancies for the Fe 4*b* octahedral site in γ-Fe_2_O_3_ was established that will possibly have a strong influence on their local surroundings, which will be discussed further in the text [21]. Moreover, the findings from synchrotron PXRD confirmed the composite nature of the MND sample with 98.89 and 1.11% weight fraction of ND and γ-Fe_2_O_3_ phases, respectively, which could be otherwise quite difficult to identify using the usual XRD technique.

### 3.2. Magnetic Properties

**Figure 2:**
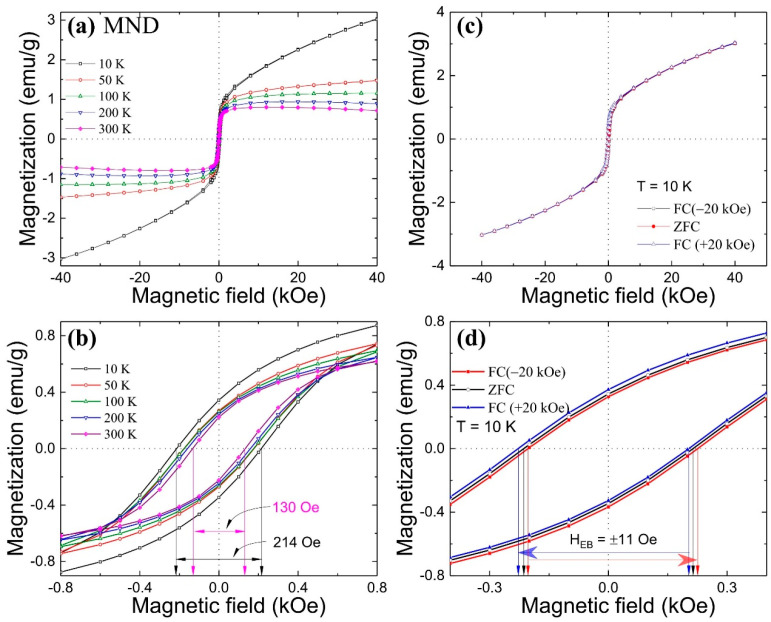
(**a**) zero field cooled (ZFC) M(H*_a_*) loops measured at different temperatures from MND, (**b**) Magnified ZFC M(H*_a_*) loops near zero-field. (**c**) ZFC and FC (±20 kOe) M(H*_a_*) loop measured at 10 K. (**d**) Magnified ZFC and FC M(H*_a_*) loop near zero-field.

The ZFC and FC magnetic hysteresis M(H*_a_*) loop measurements were carried out at different temperatures. During the ZFC measurement, the sample was initially cooled from 350 K to the desired temperature in a zero magnetic field, whereas in the case of FC, cooling was done from 350 K to the desired temperature in an external magnetic field of H*_a_*. Figure 2a shows the ZFC M(H*_a_*) loops measured in ±40 kOe field at different temperatures within the 10 K to 300 K region from the MND sample. At 10 K, a strong non-ferromagnetic signal adding to the saturation behavior of the M(H*_a_*) curve can be seen in the high-field region. Similar behavior has also been reported from dextran-coated 10 nm γ-Fe_2_O_3_ nanoparticles and was assigned to a progressive increase in the net ferrimagnetic moments with the field [21]. However, in the MND sample, it appears that the high magnetic field non-ferromagnetic signal may have possibly originated from the ND component (see Figure S1 in the supporting information). As the measuring temperature increases, the saturation magnetization decreases, and above 100 K, a strong diamagnetic signal from the ND component begins to dominate, resulting in a downward curvature. Moreover, the M(H*_a_*) measurement shows that MND retains RT magnetic behavior, which is important from an application point of view. Figure 2b shows the magnified symmetric M(H*_a_*) loop near zero-field. The coercivity (H_C_) of the MND sample drops from 214 Oe to 130 Oe on an increase of temperature from 10 to 300 K (H_C_ of ND-w 155.5 Oe at 10 K and 11 Oe at 300 K). The value of H_C_ is very near to the reported value of 250 Oe from dextran-coated and much smaller than the value of 450 Oe from uncoated 10 nm γ-Fe_2_O_3_ nanoparticles at 5 K [21]. The significant difference in the value of H_C_ is attributed to substantial magnetic interparticle interactions in uncoated nanoparticles due to the agglomeration effect that is responsible for the SSG behavior.

The finite-size effect in γ-Fe_2_O_3_ nanoparticles can lead to exchange bias (EB) coupling between the ferromagnetically aligned core spins and disordered frozen surface spins, resulting in surface SG behavior [23]. To investigate the EB phenomenon, FC M(H*_a_*) loop measurements were carried out at 10 K with a cooling field of ±20 kOe (Figure 2c). In Figure 2d, a comparison between magnified ZFC and asymmetric FC (±20 kOe) M(H*_a_*) loops near a zero magnetic field is shown. The FC M(H*_a_*) loop exhibits a loop shift in either direction depending on the direction of the cooling field with a similar EB field H_EB_ of 11 Oe. The obtained value of the H_EB_ is much smaller than the reported value of around 77 Oe under a cooling field of 50 kOe from 6.1 nm SG like γ-Fe_2_O_3_ nanoparticles [23]. The origin of a relatively small EB field from the MND sample possibly lies at the interfacial spin-exchange interaction between vacancy-induced short-range magnetic clusters and compensated ferrimagnetic γ-Fe_2_O_3_.

**Figure 3:**
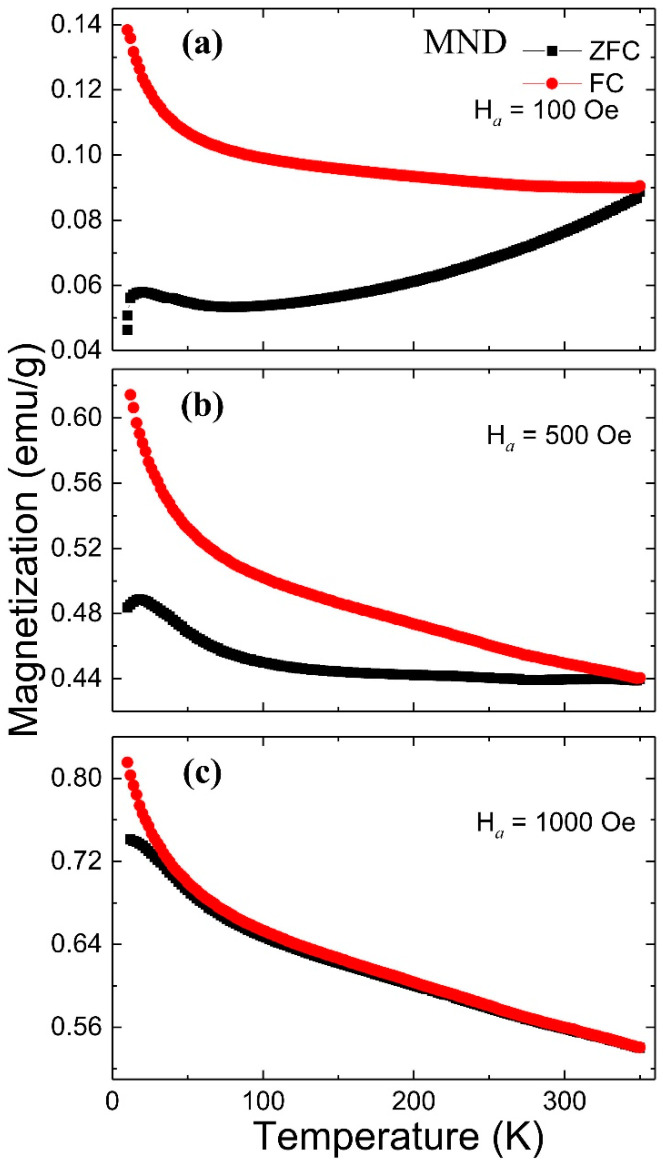
ZFC-FC temperature-dependent magnetization curve from MND measured at (**a**) 100 Oe, (**b**) 500 Oe, and (**c**) 1000 Oe.

Figure 3 shows the ZFC and FC temperature-dependent magnetization M(T) curves measured from 10 to 350 K at different H*_a_* of 100, 500, and 1000 Oe (top to bottom). In the ZFC measurements, the sample was initially cooled from 350 to 10 K without applying any H*_a_*. After reaching 10 K, an H*_a_* was applied, and the magnetic moments were recorded as the temperature increased. In the FC measurements, the sample was again cooled from 350 to 10 K under H*_a_*, and then the magnetic moments were recorded as the temperature increased. At 100 Oe, the ZFC and FC curves remain separated up to 350 °C, suggesting blocking temperature (T_B_) could be lying above it and exhibiting the typical blocking process of an assembly of SPM particles with a distribution of T_B_. As the H*_a_* increases, the separation between the ZFC-FC curve decreases. At a sufficiently high field of 1000 Oe, the ZFC-FC curve exhibits an almost overlapping behavior down to ~50 K. Furthermore, at 100 Oe, a broad, low-intensity hump can be seen around 20 K from the ZFC curve, and as the external magnetic field increases to 1000 Oe, it shifts to below 10 K featuring a blocking effect. The magnetic nanostructure that undergoes the low-temperature blocking cannot be either γ-Fe_2_O_3,_ which has a T_B_ far above 350 K, or the non-magnetic ND (Figure S2). As a consequence, we infer that at low temperatures, the interactions among the vacancy defects at 4*b* sites may have resulted in the formation of short-range magnetic clusters whose magnetic moments are thought to be collectively responding to the magnetic field. These cluster moments undergo blocking in the low-temperature region, suggesting high stoichiometric defects that can be correlated with the observed deficiency at Fe 4*b* site from PXRD. Besides this, a monotonic increase in the magnetization with the decrease of temperature can be seen from the FC curve. Contrary to the above in a few reports, a plateau below a critical temperature (in the low-temperature region) has been reported from pure γ-Fe_2_O_3_ and Fe/γ-Fe_2_O_3_ nanostructures ascribed to collective freezing of the system leading to the appearance of an SG-like phase [4,5,23,24]. Whereas, a similar temperature dependency of the FC magnetization curve was reported from dextran or SiO_2_ coated γ-Fe_2_O_3_ nanoparticles and γ-Fe_2_O_3_-alginate nanocomposite retaining in SPM state, possibly due to reduced or absence of interparticle interactions [6,25,26].

**Figure 4:**
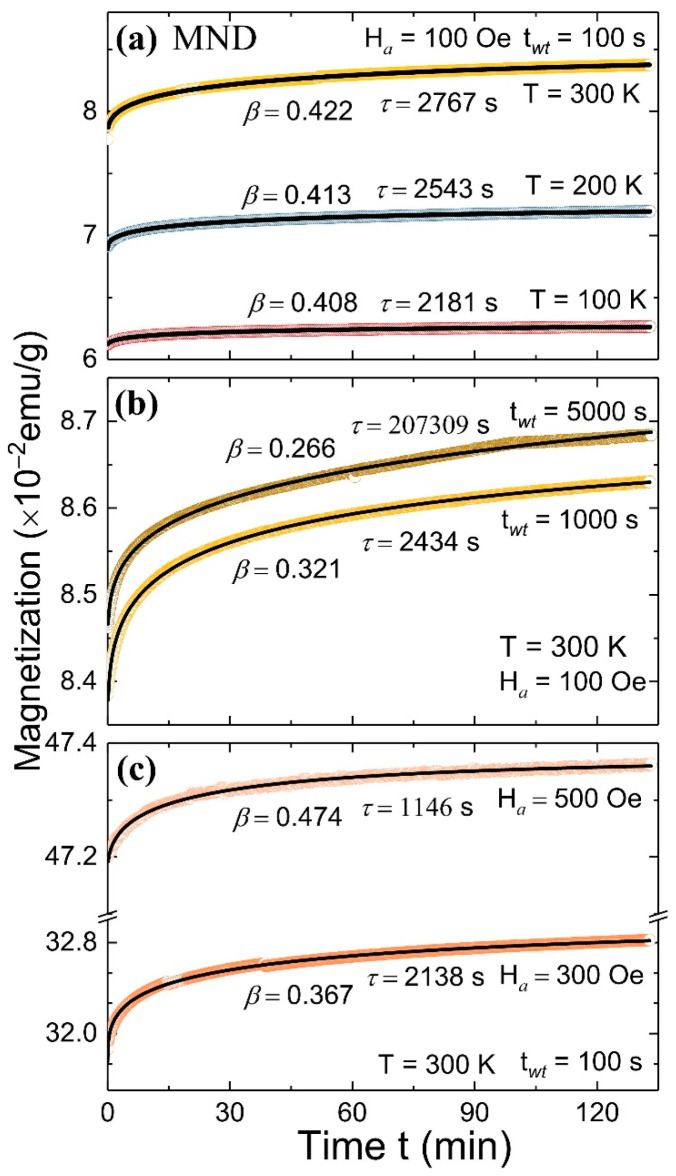
Time dependent magnetic moment relaxation curve from MND measured at (**a**) different temperatures (H*_a_* = 100 Oe, t*_wt_* = 100 s), (**b**) with different waiting time (H*_a_* = 100 Oe, T = 300 K) and (**c**) at different magnetic field (T = 300 K, t*_wt_* = 100 s).

Further insights into the interparticle interactions can be gained from the measurement of the magnetic moment relaxation process. A measurement of the time dependency of the magnetic moment relaxation M(*t*) from the MND sample was carried out at various measuring temperatures (T*_m_*), magnetic fields (H*_a_*), and different waiting times (t*_wt_*). For each relaxation curve, the sample was initially cooled in a zero magnetic field from a reference temperature of 350 K to a T*_m_* and then kept at T*_m_* for the t*_wt_*. After a lapse time of t*_wt_*, a H*_a_* field was applied, and the M(*t*) curve was recorded (Figure 4). The solid line represents the best fit to the M(*t*) curve (dots) using the stretched exponential function $M\left(t\right)=M_{0}-M_{e}\exp\left\{-{\left(t/\tau \right)}^{\beta }\right\}$, where *β* is a stretching component, $M_{0}$ an intrinsic magnetic component, $M_{e}$ glassy component, and τ characteristic relaxation time [27]. The corresponding fitted values of *β*, *τ*, and the measuring conditions are depicted in the respective figures. The value of *β* lies between 0 and 1, such that for *β* = 1 system relaxes with a single time constant (i.e., uniform energy barrier), and for *β* < 1 system involves activation against multi magnetic anisotropy energy barriers. For fixed t*_wt_* = 100 s, and at H*_a_* = 100 Oe, the values of (*β*, *τ*) exhibited slight increment from (0.408, 2181 s) to (0.422, 2767 s) with the increase of T*_m_* from 100 to 300 K, respectively (Figure 4a). Whereas, for fixed T*_m_* = 300 K, and at H*_a_* = 100 Oe, the value of *β* dropped from 0.422 to 0.266, whereas an increment in the *τ* from 2767 to 207,309 s was evident with the increase of t*_wt_* from 100 to 5000 s, respectively (Figure 4b). The enormous increment in the *τ* with t*_wt_* manifests the stiffening of the spin relaxation or the aging effect [28]. Furthermore, for fixed T*_m_* = 300 K, and at t*_wt_* = 100 s, the values of *β* show a non-monotonous dependency on H*_a_*, whereas the value of *τ* drops from 2767 s to 1146 s with the increase of H*_a_* from 100 to 500 Oe (Figure 4c). Overall, at 300 K (depending on the H*_a_* and t*_wt_*), the value of *β* varies between 0.266 to 0.474, which is well within the range reported from different SPM systems [29,30]. Moreover, *β* < 1 signifies that the MND system involves multiple anisotropy energy barriers.

For a further better understanding of the magnetic moment relaxation process of the system involving interparticle interactions or SG-like state, the M(*t*) curves were analyzed using the theoretical model proposed by Ulrich et al. [31]. According to a model for a system involving dipole interactions, the rate of decay of magnetic moment relaxation W(t)=−(d/dt)lnM(t) follows a power law $W\left(t\right)=At^{-n}$ after a lapse of a crossover time t_0_, where A is a constant, and the exponent *n* is a function of T*_m_*, H*_a,_* and particle density. *n* ≥ 1 corresponds to dense and 2/3 for weakly interacting, diluted systems having a distribution in the particle size. For MND, irrespective of measuring conditions, the fitted value of *n* to the rate of decay of M(*t*) curves remains close to zero, suggesting the absence of any interparticle interactions. Therefore, the above magnetic field, temperature, and time-dependent magnetization measurements suggest that in ND/γ-Fe_2_O_3_ composite, ~99% weight fraction of ND nanoparticles may have significantly reduced the interparticle interaction within ~1% weight fraction of γ-Fe_2_O_3_ nanoparticles such that it acts like a non-interacting SPM system having a wide distribution in T_B_ [32].

### 3.3. Magnetic Memory Effect

**Figure 5:**
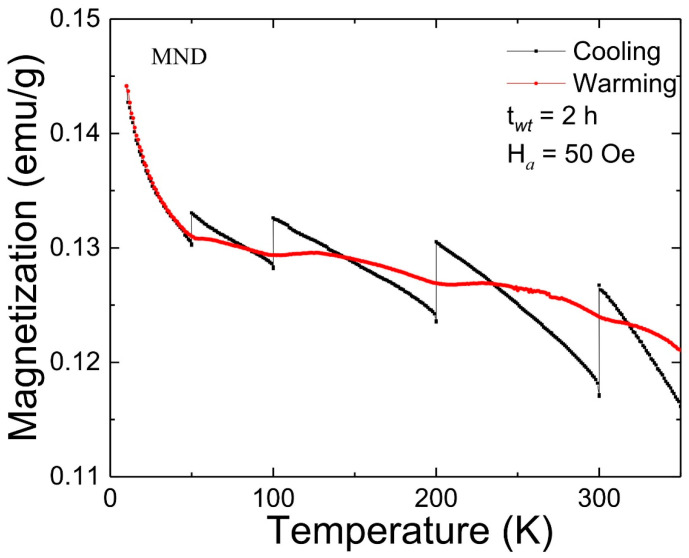
FC magnetic memory effect (MME) from MND measured at 50 Oe.

The FC MME test on MND was carried out using a protocol suggested by Sun et al. in a temperature region from 10 to 350 K [1]. Initially, the FC magnetization curve was recorded at H*_a_* = 50 Oe with sporadic stops at temperatures (T_S_) 300, 200, 100, and 50 K for a t*_wt_* of 2 h in zero-field (coined as cooling). The magnetic relaxation in zero-field at various T_S_ resulted in the appearance of the step-like magnetization curve. Subsequently, a magnetization curve was recorded while warming the sample at the same H*_a_* (coined as warming). Surprisingly, a step-like magnetization curve was reproduced around each T_S_ such that the warming curve regains its FC cooling value above it. Figure 5 depicts the FC MME response from MND. The unique part of a step-like magnetization curve is the increase in the magnetization with the decrease of temperature following reported MME from a non-interacting SPM system [2]. To further examine the interparticle interactions in MND, ZFC MME measurements were carried out (data not shown). However, from careful repeated experiments, we confirmed that ZFC MME is absent in the MND sample. The observed results differ significantly from the literature in which both FC and ZFC MME were reported from γ-Fe_2_O_3_ and Fe/γ-Fe_2_O_3_ nanostructure, confirming SG state [4,5,23]. However, the obtained MME from MND partially agrees with weakly interacting γ-Fe_2_O_3_-alginate nanocomposite from which only FC MME (but with decreasing magnetization) without ZFC MME was reported in the low-temperature region far below RT [6]. These findings further confirm the non-interacting nature of SPM γ-Fe_2_O_3_ nanoparticles in MND.

**Figure 6:**
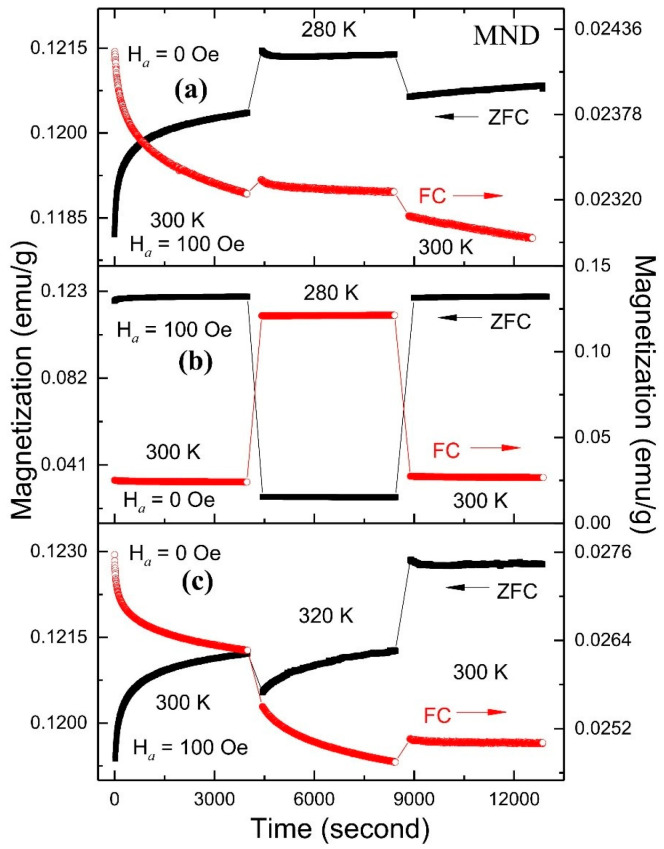
The effect of temperature-cooling (**a**) without, (**b**) with field switching, and (**c**) temperature-heating cycle without field switching during ZFC and FC magnetization relaxation from MND.

The RT MME from the MND sample was further investigated by examining the effect of field switching (0 to 100 Oe) and cooling (280 K) and heating (320 K) temperature cycles with ZFC and FC M(*t*) protocols (Figure 6). During FC (ZFC) M(*t*), the MND sample was initially cooled down from 350 K to 300 K at H*_a_* = 100 Oe (0 Oe), and the M(*t*) curve was recorded for 4000 s at 0 Oe (100 Oe); subsequently, MND was cooled to 280 K in the same H*_a_*, and the M(*t*) curve was recorded over 4000 s; lastly, MND was warmed back to 300 K in the same field, and the M(*t*) was recorded for 4000 s. It can be seen that the M(*t*) curves recorded at 300 K during the 1st and 3rd steps are in continuation even after a temporary period that the sample was cooled to 280 K (Figure 6, top panel). A similar phenomenon also appeared from ZFC-FC M(*t*) curves when the field was switch to 0 and 100 Oe during the 2nd step with the temporary cooling at 280 K, respectively (Figure 6, middle panel). The above two measurements confirm that the M(*t*) value returns to the previous state when the temperature and field are returned to the initial condition at 300 K and in 100 Oe. However, ZFC and FC M(*t*) do not restore their initial state before the temporary heating at 320 K, demonstrating no MME (Figure 6, bottom panel). The observed asymmetric response concerning temperature changes follows a hierarchical model proposed for an interacting particle system [33]. The above model is also applicable to the non-interacting SPM system in which the multi-distribution in energy barriers is originating from the particle size distribution [6].

## 4. Conclusions

We report RT ferromagnetic properties with an enhanced T_B_ > 350 K in MND. The structural investigation carried out using synchrotron PXRD confirms the composite nature of MND having ND (99% weight fraction) and γ-Fe_2_O_3_ (1% weight fraction) phases with a grain size of ~5 and 43 nm, respectively. The magnetic field, temperature, and time-dependent magnetization measurements confirm the non-interacting nature of SPM γ-Fe_2_O_3_ nanoparticles in MND having a wide distribution in T_B_. The RT-MME accomplished in MND is mediated through the multi-distribution of energy barriers originating from the distribution of γ-Fe_2_O_3_ particle size, which is interesting from both the fundamental and application points of view. The outcome of this study is technologically attractive for the future development and fundamental understanding of RT ferromagnetism in composite MND to facilitate the possible integration of ND-based spintronic devices.

## Figures and Tables

**Table 1:** Summary of the Rietveld refined PXRD fitting parameters obtained from MND and ND-w samples. The atomic positions: Fe 12*d* (x = 0.125 Å, y = 0.369(6) Å, z = 0.881(6) Å); Fe 8*c* (x = y = z = 0.975(3) Å); O 8*c* (x = y = z = 0.875(16) Å); O 24*e* (x = 0.117(15) Å, y = 0.096(15) Å, z = 0.380(18) Å).

Parameter		MND		ND-Wash
Phase		ND	γ-Fe_2_O_3_	ND
*a = b = c* (Å)		3.5729(12)	8.3468(14)	3.5579(6)
V (Å^3^)		45.612(46)	581.5(3)	45.037(22)
C	(x = y = z) (Å)	0.8301(88)	-	0.8203(8)
	Occupancy	0.214(4)	-	0.219(1)
Fe 4*b*	(x = y = z) (Å)	-	0.625	-
	Occupancy	-	0.717(203)	-
ρ (g/cm^3^)		2.9979	5.1044	3.1021
Weight fraction (%)		98.89	1.11	100
Grain size (nm)		4.74	43	4.90
wR (%)		2.77		1.69
GOF		0.25		0.35

## Supplementary Materials

The following are available online at https://www.mdpi.com/2079-4991/11/3/648/s1, Figure S1: ZFC M(H*_a_*) loops from ND-w; Figure S2: ZFC-FC temperature-dependent magnetization curve from ND-w.
