# Supplementary material for: Room Temperature Magnetic Memory Effect in Nanodiamond/γ-Fe2O3 Composites
Source: Nanomaterials (Basel). 2021 Mar 7;11(3):648. doi: 10.3390/nano11030648 (PMC8001642; doi:10.3390/nano11030648)

Supplementary data

Room Temperature Magnetic Memory Effect in Nanodiamond/γ-Fe_2O_3 Composites

Ashish Chhaganlal Gandhi, Rajakar Selvam, Chia-Liang Cheng and Sheng Yun Wu*

Department of Physics, National Dong Hwa University, Hualien 97401, Taiwan.

Figure S1. **(a)** Full ZFC M(H$_a$) loops and **(b)** magnified ZFC-M(H$_a$) loops near zero-field measured at 10 K and 300 K from ND-w.

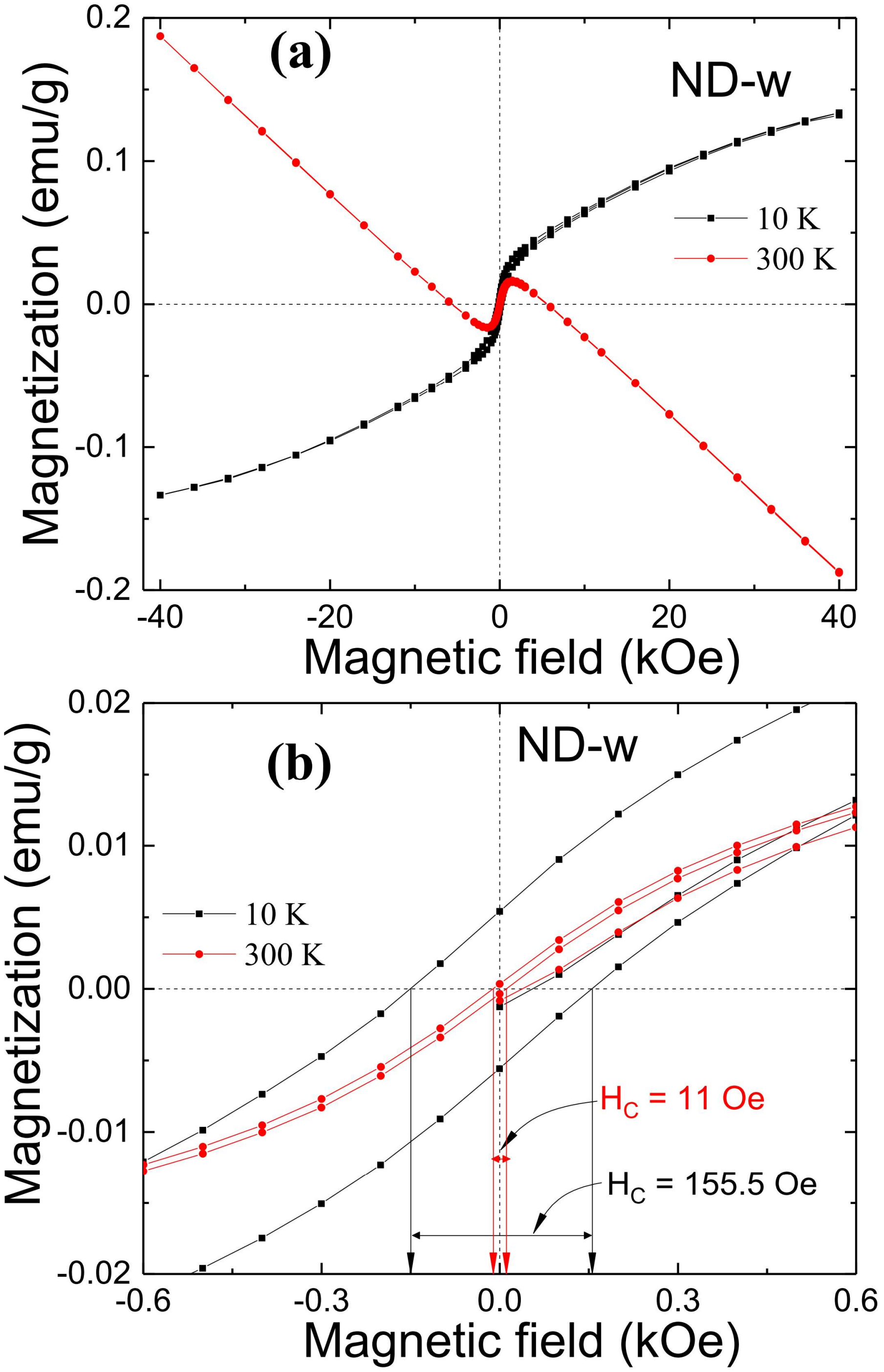

Figure S2. ZFC-FC temperature dependent magnetization curve from ND-w measured at 100 Oe.

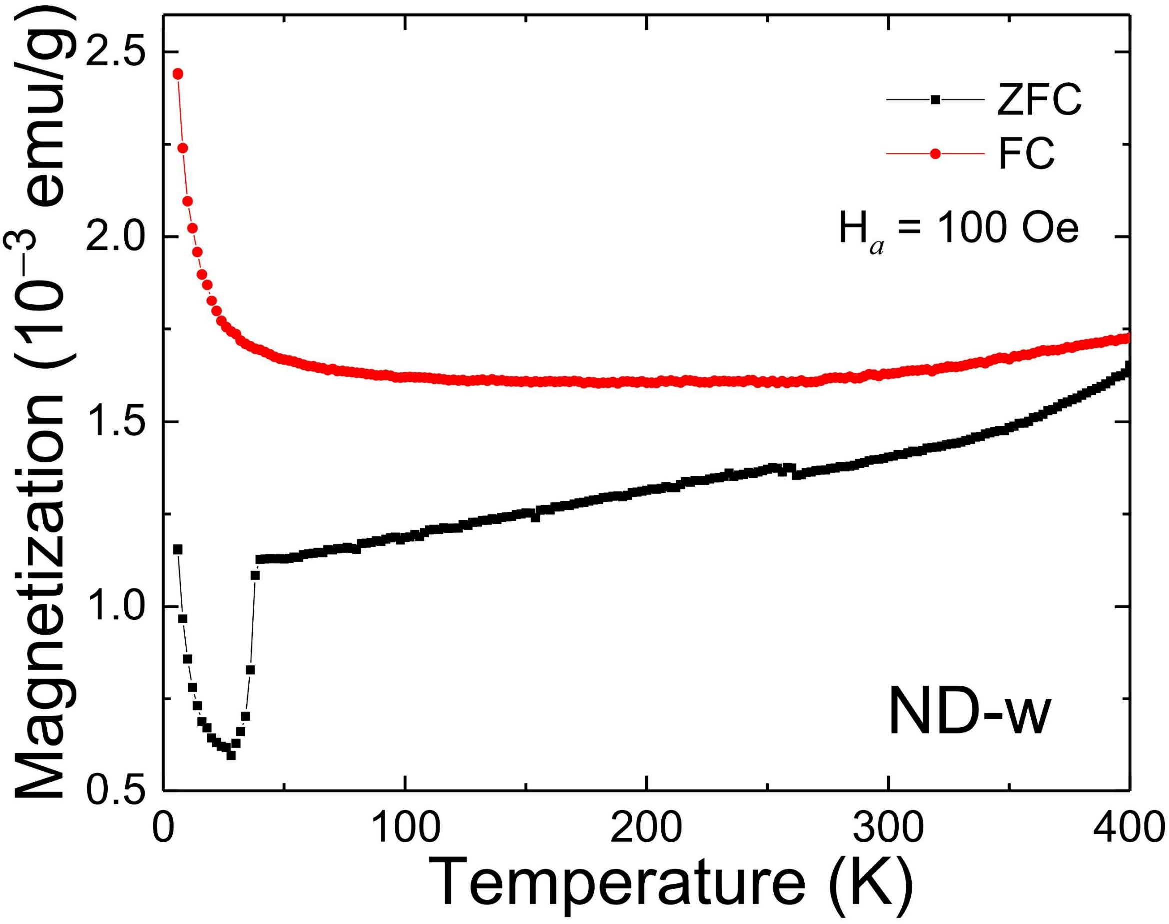

Supplement: Supplementary file 1 [file nanomaterials-11-00648-s001.pdf]
